# Effects of high-intensity interval training in combination with detraining on mental health in women with polycystic ovary syndrome: A randomized controlled trial

**DOI:** 10.3389/fphys.2022.948414

**Published:** 2022-09-29

**Authors:** Isis K. Santos, Gabriel S. Pichini, Carlindo Daniel d. Ferreira, Pedro B. Dantas, Rodrigo A. V. Browne, Victor de Queiros, Gustavo M. Soares, Ana K. Gonçalves, Breno G. Cabral, Tecia Maria O. Maranhão, Paulo Moreira S. Dantas

**Affiliations:** Federal University of Rio Grande do Norte, Natal, Brazil

**Keywords:** aerobic exercise, lifestyle, detraining, women, mental health

## Abstract

This study aimed to investigate the effects of high-intensity interval training (HIIT) and detraining on the quality of life and mental health of 23 women with polycystic ovary syndrome (PCOS). Participants were randomly assigned to the HIIT group (*n* = 12) [26.0 ± 3.92] and the control group (*n* = 11) [26.6 ± 4.68]. HIIT sessions comprised 40–60 min, 3 days a week for 12 weeks, followed by detraining for 30 days. We assessed the quality of life using the Short Form Health Survey (SF-36) and mental health by the Depression, Anxiety, and Stress Scale (DASS-21), and we compared group changes on these variables at three time points: 1) at baseline, 2) after 12 weeks of HIIT (or no training), and 3) after 30 days of detraining (or no training). The participants were classified as overweight and had a high percentage of body fat (41.5%) and irregular menstrual cycles (amenorrhea) (66.7%). Throughout training, participants in the HIIT group reported improvements in domains of the quality of life: functional capacity (M = 80.4 ± 3.4 vs. M = 87.0 ± 3.1), physical role functioning (M = 72.5 ± 9.4 vs. M = 81.8 ± 9.7), and general health perception (M = 48.6 ± 4.6 vs. M = 69.0 ± 5.8). Regarding anxiety symptoms (M = 6.4 ± 1.6 vs. M = 3.7 ± 0.7) and depression symptoms (M = 6.7 ± 1.6 vs. M = 3.8 ± 0.9), those reduced significantly after HIIT. After a 30-day detraining period, there was an increase in the significant change in the quality of life; however, domains of mental health showed instability. In summary, the HIIT program promoted improvements in the quality of life and mental health in women with PCOS. The 30 days of detraining changed the benefits in the quality of life and stability in the changes in mental health domains.

## 1 Introduction

Polycystic ovary syndrome (PCOS) is an endocrinopathy disorder that presents with several different clinical manifestations or phenotypes without a defined cause or etiology. The prevalence of PCOS in women of reproductive age has been estimated to be 5–10% (Bozdag et al., 2016). Women with PCOS have an increased prevalence of insulin resistance, hypertension, and obesity; these factors are an alarming risk for the development of metabolic changes (Moran et al., 2015). In addition, high androgen levels can result in hirsutism and acne, so these women are also more likely to experience negative feelings of depressive symptoms (Cooney et al., 2017). Depressive symptoms are likely related to a reduction in the health-related quality of life (HRQoL) from PCOS that directly affects the mental health and overall well-being of women with this disorder (Costa et al., 2018; Yin et al., 2021). This population is more prone to depression and anxiety, based on a study where women with PCOS tend to experience depressive symptoms compared to women without PCOS (Barry et al., 2011).

Given these contexts, research advances suggest that exercise is a non-pharmacological form of treatment for PCOS; the benefits of exercise have been summarized and indicate positive effects in controlling these negative factors and implications of PCOS (Moran et al., 2011; Teede et al., 2018). Based on the guidelines, lifestyle intervention (exercise + diet) is recommended; however, there are different exercise protocols performed for this population. Aerobic exercises are described more frequently and have wider applicability to different contexts (dos Santos et al., 2020; Patten et al., 2020; Santos et al., 2021). Positive changes related to clinical and metabolic exercise among women with PCOS have included improved control over glycemic levels, reduced adipose tissue, and improved depression and anxiety symptoms (Bruner et al., 2006; Thomson et al., 2016; Lopes et al., 2018; Stepto et al., 2019).

Based on different protocols, high-intensity interval training (HIIT) is described as variations between short periods of high-intensity exercise (>80–95% of maximal oxygen uptake) alternating with periods of active rest (Buchheit and Laursen, 2013a) and has shown positive improvements in populations with marked clinical conditions (Keating et al., 2017), reducing cardiovascular disease risk and metabolic parameters, thus demonstrating its possible applicability in PCOS. Recent studies have investigated and indicated beneficial effects of HIIT on the metabolic profile, reproductive parameters, body composition, and psychological well-being in women with PCOS (Almenning et al., 2015; Aktaş et al., 2019; Samadi et al., 2019).

However, when this lifestyle practice is interrupted/discontinued, the body can minimize all adaptations from exercise; this factor is known as a detraining factor. The concept of detraining is the partial or complete loss of training—induced as a consequence of the reduction or cessation of training. This process is considered a pause in physical activity and has been described for its ability to influence partial or total loss of benefits caused by exercise (Mujika and Padilla, 2000). Current studies suggest that the period of detraining after an exercise program as early as 4 weeks could be significant in reducing all improvements (Modaberi et al., 2021). Nevertheless, 2 or 4 weeks of detraining could reduce the benefits of exercise programs. Further research is needed to investigate whether higher aerobic exercise intensities induce minimal losses after detraining.

Some studies have observed that in different population groups, metabolic and functional adaptations induced by exercise programs are reduced after short periods of detraining (Leitão et al., 2019; Sakugawa et al., 2019; Rosa et al., 2020).

In particular, despite these findings, there is little evidence on how mental health parameters of women with PCOS may adjust after the HIIT protocol and a period of partial or total interruption of the exercise program. Therefore, the main purpose of this study was to analyze the effect of high-intensity interval training (HIIT) and detraining on the quality of life and mental health of women with polycystic ovary syndrome.

## 2 Methods

### 2.1 Study design

This pilot randomized clinical trial was conducted following the guidelines of the Consolidated Standards of Reporting Trials (CONSORT). The study was carried out at the Physical Education Department of the Federal University of Rio Grande do Norte, Brazil. This study was carried out in accordance with the Declaration of Helsinki, approved by the Institutional Ethics Committee under the reference number (1.863.259), and registered in the Brazilian Registry of Clinical Trials (ReBEC) www.ensaiosclinicos.gov.br (ID: RBR6nhy6h; UTN no: U1111-1204-0043). All subjects agreed to participate and signed the free and informed consent forms to be blinded, according to our previously published protocol (Dos Santos et al., 2022).

### 2.2 Participants

All participants were recruited between January 2018 and June 2019 at the Gynecology and Endocrinology Clinic of the Januario Cicco Maternity School during medical consultations, as well as on social networks, and through the Integrated Academic Activities Management System (SIGAA). PCOS was diagnosed according to the Rotterdam criteria, with a minimum of two of the following three criteria being apparent: 1) polycystic ovary morphology; 2) oligo/amenorrhea; and 3) hyperandrogenism (clinical and/or biochemical) (Eshre, 2004). The inclusion criteria were as follows: 1) aged between 18 and 40 years; 2) body mass index greater than or equal to 18.5 kg/m^2^ and less than 40 kg/m^2^; 3) no medication use for at least 3 months (i.e., oral contraceptive pills and/or metformin); and 4) no practicing physical activity and/or physical exercises in the last 6 months or sedentary (as defined by self-reporting not regularly exercising vigorously or moderately). Exclusion criteria were as follows: 1) pregnant women; 2) presenting androgen-secreting tumors, Cushing’s syndrome, congenital adrenal hyperplasia, hyperprolactinemia (greater than 40 ng/ml), and thyroid dysfunction; 3) diabetes mellitus; and 4) positive answer in the Physical Activity Readiness Questionnaire (PAR-Q) or some other contraindications for physical activity.

### 2.3 Procedures

**FIGURE 1:**
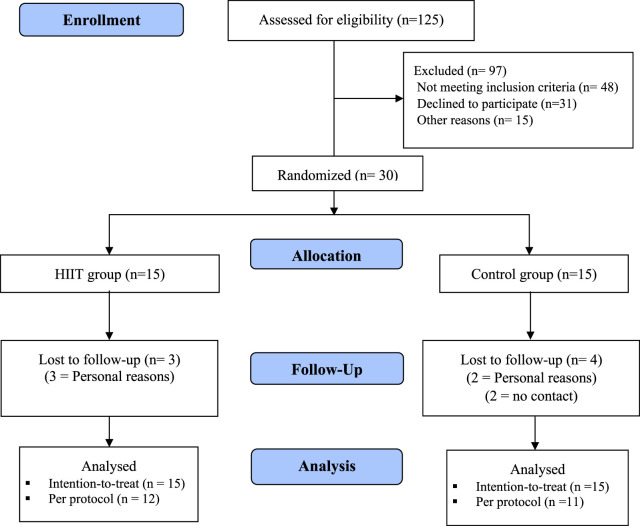
Flowchart of the study.

A total of 125 women were volunteered/recruited for participation; only 30 met the eligibility criteria for randomization. After obtaining informed consent, the participants were randomly allocated, using a table of random numbers generated by a computer (https://www.randomizer.org/), into the HIIT group and the control group in the proportion of 1:1. Participants and researchers were blinded to group assignment. The allocations were made in sealed, opaque, and numbered envelopes, kept in a locked place, and were opened by an independent administrator. The researcher was blinded during the data analysis. These participants were allocated to the HIIT group (*n* = 15), who participated in a 12-week HIIT program, or to the control group (*n* = 15), who received advice and encouragement to engage in regular physical activity, after the process; 23 women with PCOS finalized the study, Figure 1.

### 2.4 Initial screening

#### 2.4.1 Measurements

Initially, the participants underwent an initial health screening, filled out a medical history questionnaire, a Physical Activity Readiness Questionnaire (PAR-Q) (Thomas et al., 1992), and a short version of the International Physical Activity Questionnaire (IPAQ) (Craig et al., 2003). The presence of hirsutism was reported using the modified Ferriman–Gallwey score (Goodman et al., 2015) before the study. Subsequently, the participants underwent a blood sample collection after 12 h of overnight fasting (baseline), at the Clinical and Epidemiological Research Laboratory (PesqClin). The metabolic profile included fasting glucose, the oral glucose tolerance test (OGTT-75g), total cholesterol, high-density lipoprotein cholesterol (HDL-c), low-density lipoprotein cholesterol (LDL-c), triglycerides, and glucose in the blood (Cobas Mira Plus).

After this process, body weight (kg) and height (m) were measured, and the body mass index (BMI) was calculated. BMI classification was according to the criteria of the World Health Organization (WHO) (i.e., normal weight: 18.5–24.9 kg/m2; overweight: 25–29.9 kg/m2, and obesity: ≥30 kg/m2) (WHO Status, 1995). Waist and hip circumferences were measured, and the waist-to-hip ratio was calculated and classified according to the WHO criteria (i.e., ≥85 cm presents a risk of cardiovascular disease) (Organization, 2011). The measurements were performed by an experienced evaluator who followed all the recommendations of the International Society for the Advancement of Kinanthropometry (ISAK) (Silva and Vieira, 2020). Then, the participants remained in the supine position in a quiet room with a controlled temperature (23–26°C degrees) to measure the cardiac frequency at rest (FCR) through an HR monitor (Polar FT1, Polar Electro Finland Oy, Finland). Afterward, blood pressure was measured three times with an interval of 1 min by the oscillometric method (Omron^®^ HEM7200, United States). The mean of the last two measurements was considered to be resting blood pressure. Finally, body composition was measured by dual-energy X-ray absorptiometry (GE Healthcare^®^ Lunar Prodigy Advance, Madison, United States). The percentages of total fat, trunk fat, android, and gynoid were quantified.

For the main outcomes, all the participants completed the questionnaire on the health-related quality of life (SF-36) (Motamed et al., 2005) and the Depression, Anxiety, and Stress Scale (DASS-21) (Machado and Bandeira, 2013; Martins et al., 2019).

**Health-related quality of life (HRQoL):** The Medical Outcomes Study Short Form 36 (SF-36) questionnaire evaluated the concepts of quality of life measurements (Ware and Sherbourne, 1992). It is an instrument formed by items related to limitations due to health problems (physical functioning) or limitations in social activities due to physical or emotional problems (social functioning), vitality (energy and fatigue), perception of pain, general health, and mental health. The final score ranges from 0 to 100, where 0 corresponds to the worst general state of health quality and 100 to the best state. This instrument is easy to apply and has been translated into Portuguese with high reproducibility and validity (Ciconelli et al., 1999).

**Depression, Anxiety, and Stress Scale (DASS-21):** this scale was developed by Lovibond and Lovibond (1995), is based on the tripartite model, and aims to measure and differentiate as much as possible the symptoms of anxiety, depression, and stress determined by the sum of the scores of the 21 items. Using DASS-21, participants indicate the degree to which they experience each of the symptoms described in the items during the last week (previous week), on a 4-point Likert-type scale, being between 0 (not applicable) and 3 (applied) if much, or most of the time. The validated Brazilian–Portuguese version of DASS-21 was used in this study (Vignola and Tucci, 2014).

Outcome measures (the quality of life and mental health) were collected before and after 12 weeks of the protocol (72 h after the last exercise training session), and after 30 days of detraining. All tests and questionnaires were administered by experienced researchers.

### 2.5 Intervention

The HIIT protocol followed what we proposed in our previous study, which was a program that consists of a protocol that demonstrates positive affective valence during the sessions (Dos Santos et al., 2022). All HIIT (walking/running) sessions were performed on the race track (i.e., 400 m). The HIIT program included 10 weeks with a 1:3 interval/recovery ratio and the last 2 weeks with a 1:1 interval/recovery ratio. The HIIT running protocol consisted of 10 intervals of 1 min at 95% of the maximum heart rate (HRmax) interspersed with 10 active recoveries of 3 min at 70% of HRmax. This HIIT protocol was adopted because its 1:3 interval/recovery ratio makes the protocol more tolerable; in addition, its 40 min volume together with active recovery allows for a high caloric expenditure per session (Buchheit and Laursen, 2013). The exercise sessions lasted 50 min, including 5 min of warm-up at 60–65% of HRmax predicted for the age (FCmax, 220 - age), followed by 40 min of the HIIT running protocol and 5 min of relaxation (Table 1). All participants were instructed about the intensity of intervals and recovery periods. They were instructed to run at intervals and to walk during recovery so as to reach the determined intensity target. The HR was monitored continuously using an HR monitor (Polar FT1, Polar Electro Finland Oy, Finland). Attendance at the session was recorded. Patients in the HIIT group were instructed not to engage in any other physical training during the study period, and on intervention days, participants were encouraged to adhere to the study protocol through weekly reminders, phone calls, and messages on social networks (i.e., WhatsApp). All sessions were supervised by an exercise physiologist.

**TABLE 1:** Protocol of HIIT intervention during 12 weeks.

Week	Duration (min)	Interval intensity (1 min)	Recovery intensity (3 min)	Interval intensity (1 min)	Recovery intensity (1 min)
		(% HR maximum)		(% HR maximum)	
1	50	90–95	70–75		
2	50	90–95	70–75		
3	50	90–95	70–75		
4	50	90–95	70–75		
5	50	90–95	70–75		
6	50	90–95	70–75		
7	50	90–95	70–75		
8	50	90–95	70–75		
9	50	90–95	70–75		
10	50	90–95	70–75		
11	30			90–95	70–75
12	30			90–95	70–75

The control group protocol consisted of advice on the importance of regular physical activity and encouragement for the group to adhere to the recommended weekly >150 min of moderate-intensity training for 12 weeks. It was advised/requested that all participants maintain their diet normally during the intervention period, without any restriction or diet plan.

**Detraining protocol:** after the end of the HIIT protocol, participants were instructed to return to their daily lifestyle for 4 weeks (30 days) and to resume their activity level before the study started.

After 12 weeks and detraining protocol, the level of physical activity (using IPAQ) of all participants in the HIIT and control groups was assessed to know whether the recommended exercises were followed.

### 2.6 Statistical analyses

The normality of the data was verified using the Shapiro–Wilk test and z scores for asymmetry and kurtosis (−1.96 to 1.96). Descriptive data were expressed as mean ± standard deviation (SD) or absolute and relative frequency (%). We calculated the beta coefficient ( ᵝ ) and 95% confidence interval. The independent *t*-test or Fisher’s exact test was used to compare the characteristic variables of the sample between the groups. A model of a generalized estimating equation (GEE) with gamma distribution was used to evaluate the effect of group interaction overtime on the results of the quality of life and mental health. The quality of the fit of the models was verified by the normal Q-Q graph and by the Akaike information criterion. Cohen’s d was used to determine the effect size (ES) of the mean difference. Cohen’s criteria for interpreting the magnitude of the ES were followed: <0.50, small; 0.50 to 0.79, average; and ≥0.80, large (Cohen, 2013). For all analyses, *p* < 0.05 was adopted as statistically significant. Intention-to-treat and per-protocol analyses were used for data analyses. The data were analyzed using IBM SPSS Statistics for Win./v.25 (IBM Corp., Armonk, NY).

## 3 Results

Table 2 shows the basic characteristics of the participants in the groups. There were no differences between the HIIT group and the control group (*p* > 0.05). The menstrual cycle of both groups showed a higher prevalence of irregular menstrual cycles being classified as amenorrhea. The Ferriman–Gallwey score showed mild hirsutism among women in both groups. The participants were classified as overweight and had a high percentage of fat. Participants attended ≥70% of sessions. There were no adverse events among the participants involved in the HIIT protocol. There were dropouts in both groups, in which three participants dropped out of the HIIT group due to personal problems. Two participants in the control group reported personal problems, and two did not justify abandonment, and we were unable to contact them. The supplementary table presents the information.

**TABLE 2:** Baseline characteristics of the HIIT and control groups.

	HIIT group (*n* = 15)	Control group (*n* = 15)	P*
Age, years	26.0 ± 3.92	26.6 ± 4.68	0.707
Married, n (%)	7 (46.7)	5 (33.3)	0.355
Post-secondary education, n (%)	9 (60.0)	11 (73.5)	0.500
Ferriman–Gallwey score	10.7 ± 4.9	9.00 ± 5.5	0.373
Body mass, kg	76.0 ± 15.4	73.1 ± 17.4	0.630
Body mass index, kg/m^2^	28.9 ± 5.66	28.4 ± 6.69	0.818
Waist-to-hip ratio	0.79 ± 0.07	0.79 ± 0.06	0.877
Body fat, %	41.4 ± 5.86	41.5 ± 6.08	0.988
Gynoid fat, %	45.7 ± 1.7	43.6 ± 3.2	0.584
Android fat, %	45.7 ± 2.1	45.7 ± 2.1	0.971
Trunk fat, %	43.3 ± 1.7	43.0 ± 1.7	0.272
BMD, kg	1.21 ± 0.02	1.19 ± 0.02	0.323
FG, mg/dL	93.6 ± 2.9	91.5 ± 1.9	0.990
TC, mg/dL	196.3 ± 8.8	190.8 ± 9.4	0.860
HDL, mg/dL	45.9 ± 2.8	42.1 ± 1.9	0.284
LDL, mg/dL	127.1 ± 6.8	124.9 ± 7.2	0.721
TG, mg/dL	117.2 ± 17.3	119.9 ± 17.2	0.469
OGTT, mg/dL	123.9 ± 13.4	114.7 ± 7.1	0.160
Menstrual cycle status, n (%)			
Regular	6 (40.0)	5 (33.3)	0.371
Amenorrhea	7 (46.6)	8 (53.3)	
Oligomenorrhea	2 (13.4)	2 (13.4)	

Data are expressed as mean ± SD.

*p-value refers to the independent *t*-test or chi-squared test.

Abbreviations: BMD, body mineral density; HIIT, high-intensity interval training; FG, fasting glucose; TC, total cholesterol; HDL, high-density lipoprotein; LDL, low-density lipoprotein; TG, triglycerides; OGTT, oral glucose tolerance test.

Table 3 shows the results of the quality of life (HRQoL) and mental health between the groups [per protocol and intention-to-treat analyses]. The data per protocol showed there was a significant interaction and improvement in the domains: functional capacity (*p* = 0.021; ES = 1.7), physical role functioning (*p* = 0.027; ES = 1.7), general health perception (*p* = 0.021; ES = 3.1), anxiety (*p* = 0.025; ES, 1.0), and depression (*p* = 0.031; ES, 1.7) (Table 3).

**TABLE 3:** Health-related quality of life **(**HRQoL) and mental health of women with PCOS at baseline, after 12 weeks of HIIT and after 4 weeks of detraining.

	HIIT group per protocol (*n* = 12)			Control group per protocol (*n* = 11)					
	Baseline	12 weeks	Detraining	Baseline	12 weeks	Detraining	∆ (95% CI) 12 weeks–baseline	∆ (95% CI) detraining–baseline	P^*^
HRQoL									
Per protocol									
Physical functioning	80.4 ± 3.4	87.0 ± 3.1	86.1 ± 2.5	83.1 ± 3.0	84.5 ± 4.5	82.2 ± 3.6	4.0 (−7.0, 15.1)	3.4 (−2.8, 9.7)	**0.021**
Physical role	72.5 ± 9.4	81.8 ± 9.7	74.4 ± 9.3	62.5 ± 10.7	89.7 ± 9.0	76.1 ± 8.8	−32.8 (−64.6, −0.9)	16.9 (−4.6, 38.5)	**0.027**
Bodily pain	67.0 ± 4.8	61.6 ± 4.3	70.5 ± 3.1	70.7 ± 4.0	73.2 ± 5.0	70.8 ± 4.0	−10.2 (−27.5, 7.1)	13.6 (0.9, 26.2)	0.107
General health	48.6 ± 4.6	69.0 ± 5.8	62.6 ± 6.0	57.0 ± 6.8	58.4 ± 7.3	54.8 ± 5.4	17.7 (−7.3, 42.7)	0.6 (−17.2, 18.5)	**0.021**
Social functioning	84.2 ± 5.3	76.0 ± 5.6	72.2 ± 6.5	72.7 ± 7.4	75.3 ± 6.6	72.0 ± 7.1	−13.3 (−40.7, 14.1)	5.5 (−9.6, 20.6)	0.488
Emotional role	80.2 ± 13.3	75.0 ± 8.1	76.2 ± 12.2	71.2 ± 13.6	79.9 ± 11.7	61.4 ± 10.6	−30.7 (−86.0, 24.9)	14.4 (−29.2, 58.0)	0.061
Vitality	46.6 ± 5.6	57.9 ± 6.5	58.1 ± 6.4	40.9 ± 5.1	50.4 ± 6.5	45.8 ± 6.2	−7.0 −37.4, 23.0)	9.1 (−8.7, 26.9)	0.674
Mental health	59.3 ± 3.8	77.0 ± 5.2	69.5 ± 4.6	61.0 ± 4.3	67.6 ± 4,3	59.1 ± 5.0	5.1 (−13.6, 23.8)	8.7 (−11.7, 29.1)	0.213
Mental health									
Per protocol									
Stress	7.4 ± 1.8	6.0 ± 1.3	7.3 ± 1.2	8.9 ± 1.3	8.0 ± 1.0	8.6 ± 1.1	0.4 (−4.9, 5.8)	0.9 (−2.2, 4.1)	0.456
Anxiety	6.4 ± 1.6	3.7 ± 0.7	3.5 ± 0.6	5.0 ± 1.1	4.0 ± 0.6	5.4 ± 1.1	−0.5 (−5.1, 4.1)	−2.6 (−5.6, 0.4)	**0.025**
Depression	6.7 ± 1.6	3.8 ± 0.9	3.8 ± 0.7	5.1 ± 1.2	6.1 ± 1.0	7.6 ± 1.4	−2.3 (−7.2, 2.6)	-−1.0 (−4.0, 1.9)	**0.031**

Data are expressed as mean ± SD; mean difference (**∆**); 95% confidence interval (CI).

Bold values indicate significance at *p* < 0.05.

^*^ *p*-value refers to the interaction effect of groups by the time of the generalized estimating equation model.

Abbreviations: HIIT, high-intensity interval training; HRQoL, health-related quality of life.

## 4 Discussion

The main finding of this pilot randomized clinical trial was 12 weeks of HIIT. This was found to be effective in improving physical functioning, physical role, and general health perception, as well as reducing scores of anxiety and depression in women with PCOS; in addition, it was observed that after 4 weeks of detraining the QoL domains were reduced, and the mental health domains (anxiety and depression) showed stability in the HIIT group different from the control group.

Our results showed that HIIT improved QoL and mental health compared with the control group. These results show that it is extremely relevant and important for the population studied to practice exercises since scientific evidence points to the existence of a high prevalence of psychological disorders (e.g., depression, anxiety, etc.) in women with PCOS (Barry et al., 2011; Cooney et al., 2017), and it is necessary to investigate new strategies that can minimize the negative factors of PCOS.

Similar to our previous studies reported, improvement in the quality of life and mental health domains (i.e., anxiety and depression) after HIIT results in clinical benefits for this population after participating in aerobic exercise (Lopes et al., 2018). Based on evidence, manifestations caused by hirsutism, such as hair growth, the appearance of acne, and loss of female characteristics, show reduced self-esteem and negatively affect the mental health of patients (Tzalazidis and Oinonen, 2020). It has been shown that an important factor that may increase the severity of depressive symptoms in women with PCOS is that the levels of satisfaction with body image are associated with overweight, obesity, and symptoms of hirsutism, consequently increasing negative feelings in this population (Becker et al., 2019; Kogure et al., 2020).

Previous studies have supported the superior health benefits of vigorous exercise compared to moderate exercise in the PCOS population, which, however, is clear in the literature about the effectiveness of aerobic training in managing clinical improvements in the health parameters in women with PCOS population. In addition, the lack of a combined intervention (i.e., HIIT + diet) and questions involving the different phenotypes of PCOS (A-D) may have interfered with the responses to physical exercises (Woodward et al., 2019; Patten et al., 2020).

In addition, studies have suggested that HIIT programs can improve physiological functions and reduce cardiovascular endurance in middle-aged women. Based on evidence, exercises stimulate the synthesis and circulation of serotonin in the bloodstream (Jafari et al., 2018; Irandoust and Taheri, 2019).

Furthermore, the results clarify some gaps involving the deleterious effects of detraining, showing that although the intervention with HIIT has been effective in improving some areas of the quality of life for women with PCOS, after the detraining period, a substantial loss of favorable adaptations obtained during the intervention was observed. Similar to our study, Esain et al. (2017) also found that after a period of detraining, there is a noticeable decline in most domains of the quality of life (SF-36 items), and this decline was more evident in women. These results reinforce the importance of maintaining physical exercise since this interruption has been associated with a reduction and decline in aerobic capacity and body strength, in addition to physical function and consequently general health (Esain et al., 2017).

However, after detraining, the mental health domains (anxiety and depression) showed stability in the HIIT group different from the control group. To likely explain this result, the endorphin hypothesis written by Steinberg & Sykes (1985) proposes that exercise is associated with the release of endogenous opiates such as beta-endorphins and, consequently, improves mood and the feeling of well-being (Steinberg and Sykes, 1985); in addition, the 12-week intervention can result in a source of distraction or an “interval” strategy of daily worries and depressing thoughts and increased self-esteem.

To the best of our knowledge, this is the first study to compare the effects of HIIT and detraining on the quality of life and mental health of women with PCOS. Another study was carried out to determine whether the favorable cardiopulmonary and metabolic benefits induced by the moderate-intensity aerobic training program are maintained after 12 consecutive weeks of interruption (detraining period) in this population. The results showed that in women with PCOS, detraining resulted in a complete loss of favorable adaptations obtained after the intervention (Orio et al., 2008).

The main limitations to this study include the inclusion of women with different PCOS phenotypes; small sample size, lack of dietary control, no report of psychiatric comorbidities, and calculation of maximal HR based on a formula to prescribe the intensities of the HIIT session instead of a parameter identified by a maximal incremental test or the use of HR reserve as recommended. Thus, we suggest that further clinical trials be developed with different subgroups based on PCOS phenotypes, with a longer duration of interventions (exercises and detraining) and intense monitoring of the participants’ food consumption.

Considering that lifestyle modification (exercise and diet) is considered the first-line therapy for overweight/obese women with PCOS, we reinforce the importance of studies that investigate different protocols expected to help this population. Thus, this study contributes to narrowing the gap in the literature on the effects of HIIT on mental health in overweight/obese women with PCOS, thus collaborating to optimize exercise prescription for this population. However, further clarification is needed on the clinical relevance of the acute effect of detraining on mental health.

## 5 Conclusion

In conclusion, our data indicate that HIIT promoted improvement in the quality of life and mental health domains in women with PCOS; in addition, it was observed that after 4 weeks of detraining there were significant losses in the quality of life and stability in mental health domains (anxiety and depression) in the HIIT group, different from the control group.

From a practical perspective, trainers may consider 1:3 interval/recovery is more tolerable for physically inactive and overweight women with PCOS as an alternative training method for enhancing psychological aspects, thus pointing out that a break in the practice of physical exercises can result in clinically important negative consequences in this group. In this regard, it is recommended that women with PCOS should always be exercising on a regular basis and that they can be monitored during treatment by a multidisciplinary team (Buchheit and Laursen, 2013b; Annagür et al., 2013).

## Data Availability

The original contributions presented in the study are included in the article/supplementary material; further inquiries can be directed to the corresponding author.
